# Enhanced Foot Proprioception Through 3-Minute Walking Bouts with Ultra-Minimalist Shoes on Surfaces That Mimic Highly Rugged Natural Terrains

**DOI:** 10.3390/biomimetics9120741

**Published:** 2024-12-05

**Authors:** Andrea Biscarini, Andrea Calandra, Alberto Marcucci, Roberto Panichi, Angelo Belotti

**Affiliations:** Department of Medicine and Surgery, University of Perugia, 06132 Perugia, Italy; andrea.calandra@unipg.it (A.C.); alberto.marcucci7@gmail.com (A.M.); roberto.panichi@unipg.it (R.P.); angelo.belotti1@unipg.it (A.B.)

**Keywords:** minimalist footwear, postural stability, stabilometry, EMG, VAS

## Abstract

The use of minimalist shoes can lead to enhanced foot somatosensory activation and postural stability but can also increase the incidence of overuse injuries during high-impact or prolonged activities. Therefore, it appears useful to explore new strategies that employ minimalist shoes to effectively facilitate the somatosensory activation of the foot while minimizing acute and cumulative joint stress and risk of injury. To this purpose, this study introduces a novel exercise paradigm: walking for three minutes in ultra-minimalist shoes on artificial flat surfaces designed to mimic highly rugged natural terrains. The activity of foot muscles and lumbar multifidus, pain perception level, and stabilometric parameters were recorded and analyzed to characterize the novel exercise, comparing it to walking barefoot or in conventional shoes on the same rugged surface. Compared to being barefoot, ultra-minimalist shoes effectively filter nociceptive stimuli from the rugged surface, while compared to conventional shoes, they enhance the somatosensory input supporting static stability. Walking with ultra-minimalist and conventional shoes yielded higher gastrocnemius activity and lower tibialis anterior and multifidus activity compared to barefoot walking. This study highlights a practical and safe framework for enhancing foot somatosensory activation and postural stability. The new intervention is suitable for people of all ages, requires minimal time commitment, and can be performed in controlled environments such as homes, gyms, and healthcare facilities.

## 1. Introduction

The study of footwear biomechanics has undergone substantial advancements over the past few decades [1]. Among the innovations in this field, minimalist shoes have emerged as a notable trend, attracting interest from athletes, researchers, physical therapists, and health enthusiasts [2,3]. Minimalist shoes are designed to minimize interference with the natural movement of the foot due to their high flexibility, low heel-to-toe drop, light weight, and low stack height, as well as the absence of motion control and stability devices [4]. Their popularity stems from the fact that, drawing inspiration from barefoot principles, these shoes aim to restore natural foot function and mimic the mechanics of both barefoot walking and running, while still providing basic protection for the feet against acute injuries, such as punctures, cuts, abrasions, burns, and infections [5].

The use of minimalist shoes can lead to several beneficial adaptations, including the increased strength and size of intrinsic foot muscles [6,7,8,9,10,11,12] and increased longitudinal arch stiffness [7,10]. Furthermore, the transition from conventional to minimalist shoes induces changes in running kinematics (decreased stride length, step duration, and flight time, along with increased stride frequency), kinetics (lower loading rates and impact forces), and muscle activation patterns (reduced activity of the tibialis anterior during the pre-activation and absorptive phases of running and increased pre-activation activity of the gastrocnemius) [13,14,15,16,17,18]. Additionally, minimalist shoes can be effective for the conservative treatment of knee osteoarthritis [19,20] and hallux valgus deformation [21].

Conventional shoes, with their elevated heels, stiff soles, cushioning, and arch support, can significantly filter the somatosensory stimuli received by the plantar surface of the foot, reducing the afferent somatosensory information originating from the vast array of mechanoreceptors located in the sole of the foot [22,23]. Somatosensory input from the sole has long been recognized for its importance in the control of postural stability and dynamic gait patterns [24,25,26,27,28,29,30,31,32]. Indeed, cutaneous afferents from the foot sole activated by skin stretch and pressure convey signals into the CNS that can interact with descending motor commands at the spinal cord level, modulate lower and upper motor neuron excitability [33,34] and vestibular reflex [35], and evoke automatic postural adjustments [36]. Furthermore, they can provide proprioceptive cues about body orientation and movement concerning the ground [37,38]. It has consistently been proposed that increasing plantar cutaneous information, as may occur when one wears minimalist shoes, could improve balance control by facilitating plantar sensory cue detection and enhancing postural responses [28,29,30,31,32,39]. Recently, Cudejko and colleagues highlighted that middle-aged and older adults [40] and individuals with a history of falls [41] exhibited greater stability while standing and walking in minimalist shoes compared to conventional footwear, regardless of the visual or walking conditions. These studies prove that minimalist footwear can improve sensory feedback from the foot, leading to better postural control and stability.

The use of minimalist shoes instead of common footwear also changes the mechanical load distribution among the lower limb joints during running, reducing knee load while increasing the load on the ankle, metatarsophalangeal joint, and Achilles tendon [42,43]. This shift may explain the incidence of overuse injuries, such as Achilles tendinopathy, plantar fasciitis, and metatarsal stress fractures, observed in minimalist shoe users [44,45]. Caution is recommended when engaging in or prescribing high-impact or prolonged activities in minimalist shoes, especially when the transition from conventional shoes has not been conducted gradually to allow the body time to adapt and avoid overuse injuries [46,47,48,49].

On the whole, the abovementioned studies highlight both the potential somatosensory benefits and the increased risk of overuse injuries associated with using minimalist shoes instead of conventional footwear. Based on this evidence, it appears advantageous to explore new strategies that leverage minimalist shoes to enhance foot plantar somatosensory information while minimizing acute and cumulative stress on lower limb joint structures, ultimately reducing the risk of injuries. Somatosensory stimuli from the foot sole can be effectively enhanced by using ultra-minimalist shoes on naturally rugged terrains. Ultra-minimalist shoes are defined as minimalist footwear characterized by the highest minimalist index [4]. Safety can be maximized by engaging in low-impact activities of limited duration in a controlled environment. We believe both these goals can be achieved by walking for a limited time with ultra-minimalist shoes on artificial flat surfaces designed to mimic highly rugged natural terrains and arranged on easy routes within protected environments such as homes, gyms, and healthcare facilities. This motor task—walking on flat surfaces with small protrusions—replicates natural movement patterns, presents minimal risk, and is not only suitable but also easily administrable to individuals of all ages, including those with conditions that do not impede normal ambulation. By using surfaces that replicate the irregularities of natural terrains, the foot can experience enhanced and more natural stimuli compared to hard, flat paved surfaces, while avoiding the risks and logistical burdens associated with using ultra-minimalist shoes in natural open environments. However, using ultra-minimalist shoes on rough surfaces can cause discomfort and pain in users not accustomed to this type of footwear [47], which could affect postural control via mechanisms related to pain perception [50]. Therefore, pain perception should also be carefully evaluated to assess the validity and feasibility of this new approach.

To enhance foot somatosensory activation while maintaining a tolerable level of pain, mild to moderate pain levels, around 3.4–3.5 mm on the visual analogue scale (VAS) [51], have been suggested [52]. These levels can effectively activate mechanoreceptors, including those in cutaneous tissues, without causing significant discomfort or impairing movement efficiency. The underlying mechanism is thought to facilitate the activation of foot mechanoreceptive pathways, which may enhance joint position awareness and balance control. In contrast, pain intensities above 6.5 cm on the VAS, classified as moderate to severe, should be avoided, as such levels can trigger protective motor responses or alter neuromuscular coordination, ultimately impairing proprioceptive function [52].

In light of the aforementioned considerations, the present study aimed to compare the electromyographic (EMG) activity of foot muscles during a 3 min walk on a flat surface that mimics highly rugged terrain in three different conditions: barefoot, in ultra-minimalist shoes, and in conventional shoes. Additionally, we aimed to compare the relevant stabilometric parameters (center of pressure (CoP) mean velocity and excursion area, and mediolateral and anteroposterior CoP standard deviation) recorded during static stability tests performed barefoot after each of the three walking sessions. Finally, we assessed the level of pain perceived during the walking sessions using a questionnaire based on the 100 mm visual analogue scale (VAS) [51].

We hypothesize that ultra-minimalist shoes significantly filter the nociceptive stimuli conveyed by the rugged surface to the sole of the foot compared to being barefoot, while enhancing the somatosensory input that controls static stability compared to conventional shoes. The approach proposed in the present study balances the benefits of facilitating somatosensory activation with the need to protect against joint and tissue stress, potentially providing a practical and feasible framework for improving foot function and overall stability in a safe and controlled manner.

## 2. Materials and Methods

### 2.1. Participants

Thirteen female and ten male participants (mean ± SD age: 34 ± 7 years, range: 21–62 years; mean ± SD height: 1.69 ± 0.08 m, range: 1.55–1.89 m; mean ± SD body mass: 66 ± 9 kg, range: 50–92 kg; mean ± SD BMI: 22.1 ± 1.2; BMI range: 19.7–24.0) were recruited for the study. The inclusion and exclusion criteria for participants are outlined in Table 1. All participants gave informed consent to their inclusion in the study, which was conducted in accordance with the Declaration of Helsinki and approved by the Ethics Committee of the University of Perugia.

### 2.2. Walking Tests

**Figure 1:**
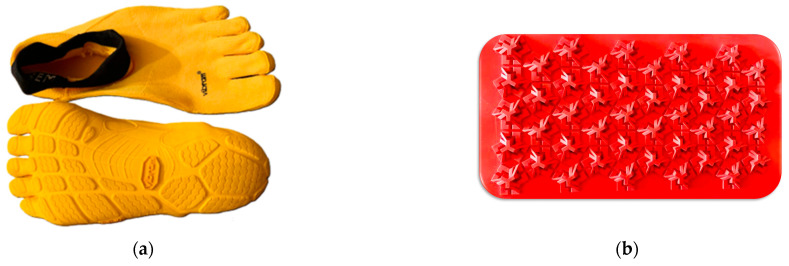
Ultra-minimalist FiveFingers^®^ shoes used in the three walking trials (**a**). Rectangular rigid slabs (35 cm length, 20 cm width, 2 cm thickness) with small, irregularly shaped protrusions simulating rough natural terrain (**b**). The slabs were arranged in series to form a straight path 10 m long.

**Figure 2:**
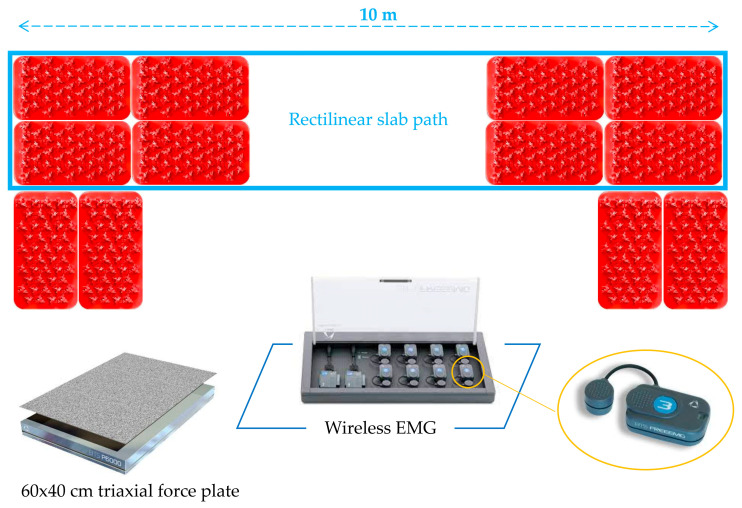
Schematic diagram of the testing area.

Walking tests were conducted in three different shoe conditions: conventional shoes, ultra-minimalist shoes, and barefoot. For the conventional shoe condition, each participant wore their own regularly used footwear. Ultra-minimalist FiveFingers^®^ shoes (Vibram, Albizzate, Italy) were distinctively characterized by their individual toe pockets, a weight ranging from 90 to 110 g (for the sizes corresponding to foot length 22.1–28.9 cm), a 2.5 mm stack height, zero heel to toe drop, high flexibility, and the absence of motion control and stability devices (Figure 1a). This gives these shoes the highest value on the minimalistic index [4]. Participants were asked to walk for 3 min, at their habitual walking cadence and velocity, on rectangular slabs made of rigid rubber (Vibram, Albizzate, Italy), each measuring 35 cm in length, 20 cm in width, and 2 cm in thickness (Figure 1b). The slabs were arranged in series to form a straight, 10 m long path (Figure 2). The surface of each slab featured small, jagged, and irregularly shaped protrusions, ranging in thickness from 0.2 to 1 cm, to simulate a rough natural terrain, such as rocky trails, uneven forest floors, gravel, or beaches with deposits of debris, small pebbles, and shells. 

At each of the two extremes of the slab line, two more slabs were placed side by side with their long axis perpendicular to the slab line to allow sufficient space for a smooth change of direction. The change of direction was performed by turning the body the opposite way around at the two extremes of the slab line, so as to render the task symmetric. During each walking trial, EMG signals were recorded from the gastrocnemius medialis (GM), tibialis anterior (TA), peroneus longus (PL), and lumbar multifidus (LM) of the dominant side of the body. EMG activity on the non-dominant side was not recorded due to the symmetrical nature of the task and because analyzing activation asymmetries between limbs was not a focus of this study.

After each walking test, participants completed a questionnaire based on the 100 mm visual analog scale (VAS) to assess their perception of regional pain in the plantar area of the foot during the test. The VAS used had major divisions, with numerical labels every 10 mm, minor divisions every 1 mm, and two descriptors representing extremes of pain intensity (e.g., no pain and extreme pain) at each end. This scale allowed for precise reporting of pain perception during the tests.

### 2.3. Stabilometric Test

Participants were asked to stand barefoot on a triaxial force platform in an open-eye condition and in a comfortable, self-chosen double-leg stance with arms alongside the body. They were instructed to stand as still as possible during the tests and to breathe normally [53]. Each test lasted for 60 s, and data acquisition started 5 s after the participant declared their readiness for testing. The flat surface of the platform was not modified for the test. All other test conditions (test room dimensions, distance of the platform from the walls, sound source, illumination, and gaze focus) adhered to the standardization criteria proposed by Kapteyn et al. [54] and Scoppa et al. [55].

### 2.4. Testing Session

**Figure 3:**
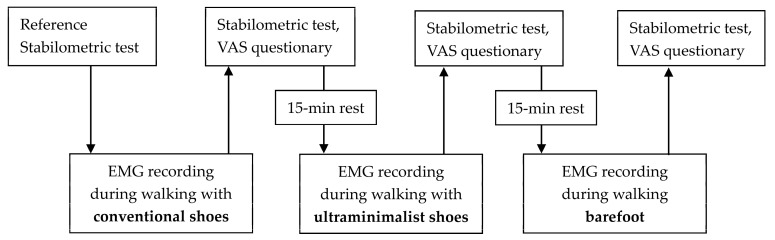
Flowchart showing the stages of the testing session.

Participants performed three walking tests in a preset order: with conventional shoes, ultra-minimalist shoes, and barefoot. A barefoot stabilometric test followed each of the walking tests, and a reference stabilometric test was performed prior to the walking tests (Figure 3). The walking test order was chosen to minimize the possible interference between subsequent tests, given the progressively increasing proprioceptive and nociceptive stimulation with the gradual reduction in the foot–ground interface. To further control and mitigate this possible interference, a 15 min rest period was allowed between successive walking trials, and a reference stabilometric test was performed prior to the walking tests. After the tests and a 5 min recovery, we recorded the EMG activity during the maximum voluntary isometric contractions (MVIC) of the selected muscles, following a procedure previously described by Biscarini et al. [56]. The EMG signals collected during the MVIC tests were utilized for offline normalization of the EMG signals recorded during the walking trials (refer to the subsequent section for specifics).

### 2.5. Data Recording and Processing

Surface EMG signals from the four selected muscles (gastrocnemius medialis, tibialis anterior, peroneus longus, lumbar multifidus) on the dominant side of the body were recorded with wireless EMG sensors (FreeEMG 1000; BTS Bioengineering, Milano, Italy) and Ag/AgCl surface electrodes placed on the muscle belly, parallel to the muscle fibers, 2 cm apart from each other. Electrodes were precisely located according to the SENIAM recommendations [57]. The raw EMG signals were differentially amplified (gain of 933), bandpass filtered (10–500 Hz), digitized (16-bit resolution, 1 kHz sampling frequency), and transformed into amplitude envelopes using a point-to-point moving root mean square filter with a 500 ms time interval. The EMG amplitude envelopes recorded from each muscle during the trials were then normalized to the highest 1 s average of the EMG amplitude envelope obtained during the MVIC of the same muscle [58]. Finally, the mean value of each normalized EMG signal was computed for statistical comparison between the task conditions.

The stabilometric parameters were obtained using a “BTS P-6000” force platform (BTS Bioengineering, Milano, Italy) with its standard flat surface. The signal recording was accomplished with a 16-bit A/D converter and a 500 Hz sampling frequency. The sway software package (BTS Bioengineering, Milano, Italy) was used for the analysis of the relevant two-dimensional CoP parameters (CoP mean velocity and excursion area) and the mediolateral and anteroposterior CoP standard deviation.

### 2.6. Statistical Analysis

An ANOVA design was used to compare data samples. The sphericity assumption was verified by the Mauchly’s sphericity test, and probabilities were corrected using the Greenhouse–Geisser and Huynh–Feldt epsilon when appropriate. The normality and homogeneity of variance between populations were assessed using the Shapiro–Wilk test and Levene’s test, respectively. When necessary to meet the ANOVA assumptions, data were transformed using the natural logarithm (ln) function. Descriptive statistics, presented as mean ± SD, consistently refer to the untransformed data, even if the analysis was performed on transformed data. EMG and VAS data samples were analyzed using a one-way repeated-measures ANOVA, with footwear condition (barefoot, ultra-minimalist shoes, conventional shoes) as a 3-level within-subject factor. Stabilometric data were analyzed using a one-way repeated-measures ANOVA, with test condition (before the walking tests and after each of the three walking tests) as a 4-level within-subject factor. The statistical power and effect size were assessed by the observed power (ω) and partial eta squared ($\eta_{p}^{2}$) coefficients, respectively, and post hoc analysis was conducted using the Bonferroni test. The significance level was set at *p* < 0.05. The SPSS software (v29) package was used for statistical calculations.

## 3. Results

### 3.1. Stabilometry

**Figure 4:**
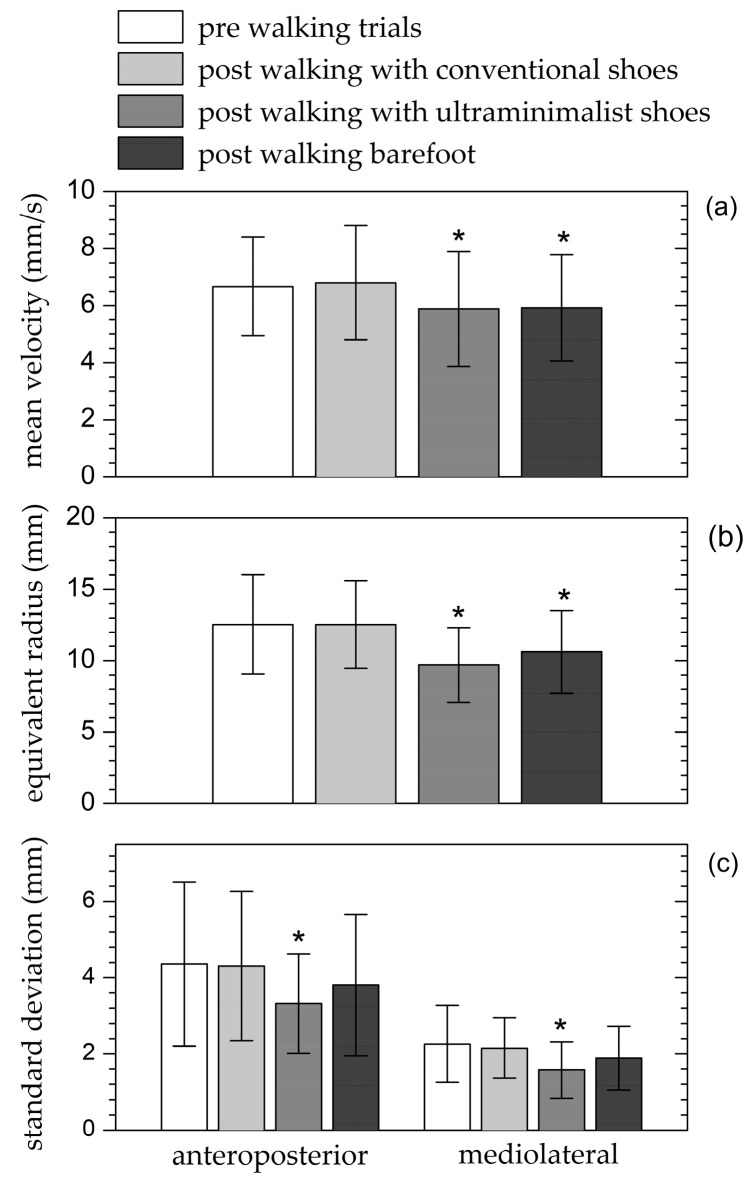
CoP mean velocity (**a**), equivalent radius (**b**), and longitudinal and transverse standard deviation (**c**) recorded during the stabilometric tests conducted before the walking trials and after each of the three walking trials (with conventional shoes, ultra-minimalist shoes, and barefoot). * Significant difference with the reference test (before walking trials).

The ANOVA results indicated that the test condition had a significant effect on all CoP parameters: velocity (p<0.001, $\eta_{p}^{2}=0.33$, ω=0.99), equivalent radius (p<0.001, $\eta_{p}^{2}=0.40$, ω=0.99), longitudinal standard deviation (p=0.030, $\eta_{p}^{2}=0.15$, ω=0.69), and transverse standard deviation (p=0.007, $\eta_{p}^{2}=0.18$, ω=0.86). Post hoc analysis demonstrated no significant differences in CoP parameters before the walking tests and after walking with conventional shoes, nor were there significant differences in CoP parameters between walking with ultra-minimalist shoes and walking barefoot. However, compared to the pre-walking test (the reference condition), CoP velocity and equivalent radius significantly decreased after walking with ultra-minimalist shoes (p<0.004) and in the barefoot condition (p<0.007), while the longitudinal and transverse CoP standard deviations significantly decreased only after walking with ultra-minimalist shoes (p=0.041 and p=0.008, respectively) (Figure 4).

### 3.2. Electromyography

**Figure 5:**
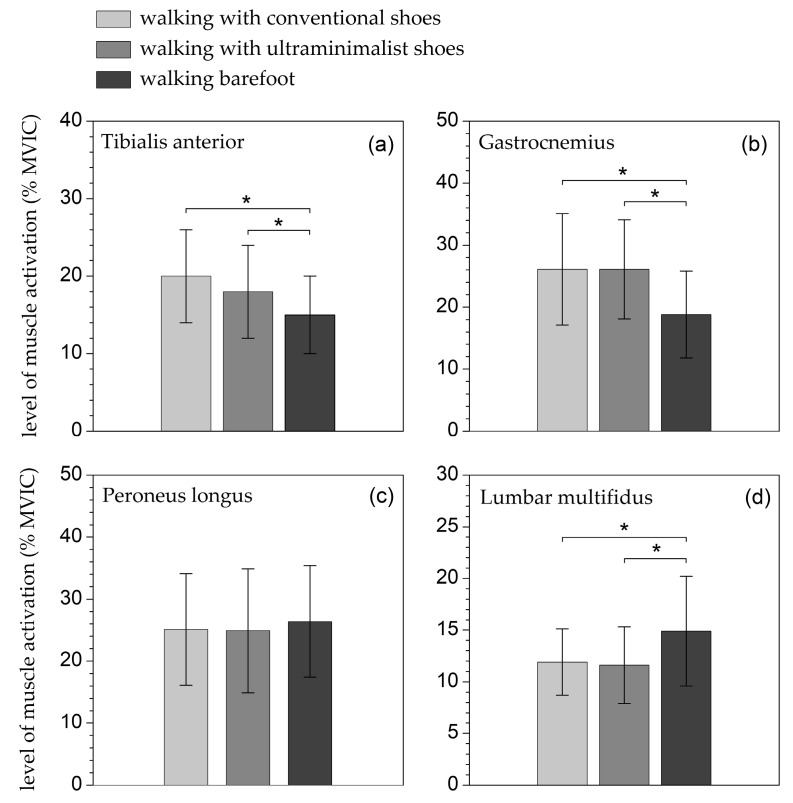
Mean level of tibialis anterior (**a**), gastrocnemius medialis (**b**), peroneus longus (**c**) and lumbar multifidus (**d**) muscle activation during the three walking trials (with conventional shoes, ultra-minimalist shoes, and barefoot). * *p* < 0.05.

The different footwear conditions significantly affected the mean EMG activity of the TA, GM, and LM muscles (p<0.001, ${0.39\leq \eta }_{p}^{2}\leq 0.54$, 0.99≤ω≤1) during the walking test but had no significant impact on the PL muscle. Post hoc analysis revealed that the mean EMG activity of each of the four muscles (GM, TA, PL, and LM) did not differ significantly when walking in conventional or ultra-minimalist shoes (Table 2 and Figure 5). However, compared with these two shoed conditions, walking barefoot resulted in a significantly lower mean EMG activity in the TA and GM (p<0.001) and a significantly higher mean EMG activity in the LM (p<0.001).

### 3.3. Regional Pain Detection

**Figure 6:**
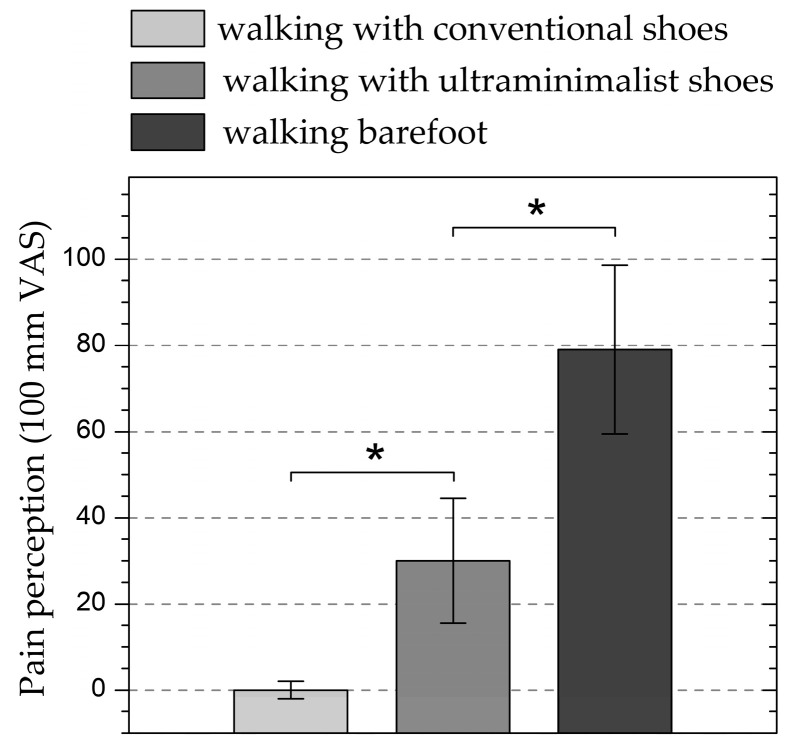
Perceived regional pain in the plantar area of the foot during the three walking trials (conventional shoes, ultra-minimalist shoes, and barefoot), assessed using a 100 mm VAS scale. * *p* < 0.001.

Detection of regional pain in the plantar area of the foot during the walking tests, assessed using the VAS scale, was strongly dependent on footwear type (p<0.001, $\eta_{p}^{2}=0.92$, ω=1). Pain perception in ultra-minimalist shoes was significantly higher than with conventional shoes (p<0.001) and significantly lower than barefoot (p<0.001). Specifically, pain perception with conventional shoes was negligible, and a difference as high as 49 mm on the VAS scale was reported between the barefoot and ultra-minimalist shoe conditions (Figure 6). This difference is significantly greater than the minimum clinically significant difference in VAS pain scores, which is approximately 10 mm in the lower range of the scale and around 30 mm in the upper range [59]. Negligible correlations (|r_s_| < 0.1, where r_s_ is the Spearman correlation coefficient) were found between participant age and the reported pain perception level within each of the three different footwear conditions.

## 4. Discussion

This study introduces a novel exercise paradigm to enhance foot somatosensory activation: walking for three minutes in ultra-minimalist shoes on artificial flat surfaces designed to mimic highly rugged natural terrains. This approach aims to retain the strong proprioceptive stimulus of barefoot walking on rugged natural terrains while providing basic protection and safety, as well as avoiding foot discomfort. The study outcomes highlight that, compared to being barefoot, ultra-minimalist shoes effectively filter nociceptive stimuli from the rugged surface, while, compared to conventional shoes, they enhance the somatosensory input that supports static stability.

Unlike walking with conventional and ultra-minimalist footwear, walking barefoot on highly rugged artificial surfaces produces a severe level of pain (Figure 5: 100 mm VAS rating of 79) [60], leading to a noticeable change in movement kinematics. The entire sole of the foot contacts the support surface in a slow and controlled manner, and the forefoot and hindfoot lift off almost simultaneously. This results in increased activation of the tibialis anterior and decreased activation of the gastrocnemius (Figure 4). Additionally, the lower limb appears stiffer, with the knee remaining semi-flexed and not fully extending, and the trunk leaning forward. This posture necessitates greater activation of the spinal erectors, which explains the increased activation of the lumbar multifidus (Figure 4). In contrast, the mild level of discomfort perceived while walking with ultra-minimalist shoes on rugged surfaces (Figure 5: 100 mm VAS rating of 30) does not result in changes in the mean level of muscle activation compared to walking with conventional shoes (Figure 4).

Contrary to other muscles, the different footwear conditions had no significant impact on the PL muscle. The reason is likely related to the fact that the slabs (Figure 1b) are stable and geometrically flat, despite being equipped with micro-reliefs, and therefore do not pose a significant challenge for maintaining balance. As a result, the musculature involved in inversion and eversion movements of the hindfoot, particularly the PL, is not substantially affected by the type of footwear.

The stabilometric data highlight improved postural stability after walking with ultra-minimalist shoes, as indicated by smaller CoP parameters (mean velocity, equivalent radius, and longitudinal and transverse standard deviation) compared to both the pre-walking test and the test conducted after walking with conventional shoes. No further decrease in stabilometric parameters was observed after barefoot walking, and participants reported a perception of foot discomfort from direct contact with the rugged surface. Possibly, contemporaneous substantial activation of both low (cutaneous) and high (pain) threshold mechanoreceptors generate conflict pathways that may deteriorate mechanisms improving postural stability. Indeed, the decreases in longitudinal and transverse CoP standard deviation after barefoot walking compared to pre-walking test values did not reach statistical significance, unlike the results observed after walking with ultra-minimalist shoes.

These findings highlight the potential of ultra-minimalist footwear walking interventions on artificial flat surfaces that mimic highly rough terrains. Such interventions can be brief, with just three minutes proving sufficient to yield beneficial effects on postural stability. They are also notably safe; unlike other exercises that stimulate the proprioceptive system, such as those performed on unstable surfaces, these interventions do not carry risks of falling or excessive mechanical strain on specific joints [61,62,63]. Additionally, they can be easily performed in controlled environments like one’s home, gyms, or healthcare facilities, without the need for expensive equipment. Suitable for individuals of all ages, including those who are deconditioned, these interventions can even be offered to those requiring assistive devices for independent walking.

In an effort to explore the potential physiological mechanisms underlying the observed outcomes, it is useful to consider the differing excitability thresholds, spatial acuity, and latency and functional significance of tactile and pain sensory signals [64,65]. Tactile receptors are sensitive to low-intensity stimuli such as light touch or pressure. They have a low excitability threshold, meaning they are easily activated by mild stimuli. These receptors are densely distributed on the skin’s surface, allowing for a greater ability to distinguish between two closely spaced points of stimulation, resulting in higher spatial acuity. Tactile information is primarily transmitted by Aβ fibers, which are myelinated, have a relatively large diameter, and conduct at high speeds (30–100 m/s), leading to lower latency. This rapid transmission enables the sensorimotor system to execute fast and accurate predictive strategies and efferent commands, such as those controlling postural stability [66,67]. As a result, the tactile receptors on the plantar surface of the foot are highly sensitive to the small irregularities of the artificial surfaces used in walking tests. 

Additionally, several studies showed that the foot sole provides cutaneous feedback about body orientation and interactions with the environment, which play a role in standing balance [68], gait [69,70], and automatic postural adjustments [36], as well as in the modulation of lower [34] and upper limb [33] muscle activity and vestibular reflexes [35]. Thus, it has been proposed that increased plantar cutaneous information could improve balance control by facilitating plantar sensory cue detection and enhancing postural responses [24,25,26,27,28,29,30,31,32,40,41].

In contrast, nociceptors, which detect potentially harmful stimuli, have a higher excitability threshold, requiring more intense stimuli to become activated [64,65]. This higher threshold prevents unnecessary pain signals in response to non-threatening stimuli. Nociceptors are also less densely packed and more evenly distributed across the skin, leading to lower spatial discrimination of nociceptive signals. Additionally, Aδ and C fibers, which carry nociceptive signals, conduct information more slowly (C fibers: 0.5–2 m/s, Aδ fibers: 12–30 m/s), resulting in higher latency. Consequently, the nociceptors on the plantar surface of the foot are less sensitive to rugged surfaces than tactile receptors and their activation can trigger protective motor responses that can alter neuromuscular coordination [52]. Moreover, it has been shown that pain stimuli on the legs or feet can cause a decrease in proprioceptive acuity and increase CoP displacement [50,71]. In addition, pain may alter spinal excitability and promote steady variations in spinal transmission that could impair motor strategies [72].

Ultimately, the thin sole of ultra-minimalist shoes filters nociceptive stimuli from the rugged surface more than tactile stimuli, maintaining the benefit of sole cutaneous activation and making undesirable pain responses ineffective. While socks may seem to offer similar benefits, their thin, stretchable fabric is easily penetrated by surface irregularities, making them ineffective at filtering nociceptive stimuli. As a result, socks are generally considered equivalent to the barefoot condition in terms of sensory experience. Additionally, during walking or running, socks tend to deform and shift relative to the foot, causing discomfort. Ultra-minimalist shoes, on the other hand, provide a structured yet thin sole that effectively reduces discomfort from nociceptive stimuli while still preserving tactile feedback, offering a more stable and comfortable experience on uneven surfaces. While this reasoning is speculative, it provides a possible framework for understanding the sensory mechanisms that may contribute to the study’s outcomes. Further studies are needed to directly investigate these hypotheses.

The primary limitation of this study pertains to the stabilometric tests, which were conducted immediately following the walking trials on rough surfaces under various footwear conditions. Consequently, the study examined only the acute effects of these walking trials on postural stability, leaving unresolved the question of how long these effects persist over time and the exact mechanisms underlying the improvement in stability. Studying the acute effects of an intervention is, however, of great interest as it provides essential insight into the immediate responses of the body. Understanding these immediate responses is crucial because it serves as a preliminary step for further research on the long-term effects. Therefore, verifying the presence of acute effects is promising for their potential persistence or cumulative impact over time and supports the need for additional studies to explore this further. Additionally, all walking trials were uniformly set to a duration of three minutes. We selected this duration as an optimal balance between providing an effective somatosensorial stimulus, minimizing cumulative joint stress, and maintaining tolerable levels of discomfort. Nevertheless, exploring trials of varying durations and comparing their effects could be valuable for optimizing the intervention protocol. This information is crucial for developing programs where ultra-minimalist footwear walking interventions on rough surfaces are periodically administered. It is hoped that future studies will address these aspects to provide further clarity.

Another limitation concerns the lack of kinematic data recording. As participants were instructed to walk at their habitual cadence and velocity, we lack the means to explore potential correlations between these variables and the study outcomes. However, clear variations in step length and cadence were observed during the trials, as participants had to change direction several times at each end of the slab path, where two additional slabs were positioned perpendicular to the main line. As a result, cadence and step length differed between the straight sections and the turns, making it challenging to draw meaningful comparisons of cadence across participants.

## 5. Conclusions

This study introduces a novel exercise paradigm for enhanced foot somatosensory activation: walking for three minutes in ultra-minimalist shoes on artificial flat surfaces designed to mimic highly rugged natural terrains. Compared to walking barefoot, the ultra-minimalist shoes effectively filter nociceptive stimuli from the rugged surface, while, compared to conventional shoes, they enhance the somatosensory input that supports static stability. This new intervention is suitable for people of all ages, requires minimal time commitment, and can be performed in controlled environments such as homes, gyms, and healthcare facilities. Ultimately, the proposed approach offers a practical and feasible framework for improving foot function and overall stability in a safe and controlled manner.

## Figures and Tables

**Table 1:** Inclusion and exclusion criteria.

Inclusion Criteria	Exclusion Criteria
- Age between 18 to 65 years; - Body mass index (BMI) within the normal range (18.5–24.9); - Foot length 22.1–24.3 cm or 25.8–28.9 cm (due to availability of ultra-minimalist shoes); - Habitual use of conventional shoes.	- Recent record of lower limb injuries; - Any form of surgery affecting balance or mobility within the last six months; - History of vestibular disorders, neurological diseases, or severe musculoskeletal conditions that could affect balance or gait; - Cognitive impairments that could interfere with test performance; - Use of medications affecting balance or motor function within the past month; - Consumption of excessive amounts of alcohol or use recreational drugs. - Pregnancy.

**Table 2:** Mean (±SD) level of tibialis anterior (TA), gastrocnemius medialis (GM), peroneus longus (PL), and lumbar multifidus (LM) muscle activation during the three walking trials (with conventional shoes, ultra-minimalist shoes, and barefoot).

	TA	GM	PL	LM
Walking with conventional shoes	20.0 ± 6.0	26.1 ± 8.5	25.1 ± 8.8	11.9 ± 3.2
Walking with ultra-minimalist shoes	18.1 ± 6.0	26.1 ± 7.7	24.9 ± 10.1	11.6 ± 3.7
Walking barefoot	14.5 ± 5.3	18.8 ± 6.6	26.4 ± 9.4	14.9 ± 5.3

## Data Availability

The raw data supporting the conclusions of this article will be made available by the authors on request.
